# Multiomics Approach Identifies Novel Genetic Determinants and Therapeutic Targets for Asthma

**DOI:** 10.1155/carj/6447989

**Published:** 2026-05-14

**Authors:** Fang Liu, Hongtao Cui, Yan Mei, Keyu Li, Yan Xu, Shuman Li, Chao Yuan

**Affiliations:** ^1^ Chongqing Traditional Chinese Medicine Hospital, Chongqing, 400021, China; ^2^ Department of Pediatrics, The First Affiliated Hospital of Chongqing University of Chinese Medicine, Chongqing, 400021, China; ^3^ Department of Pediatrics, The First Affiliated Hospital Henan University of Chinese Medicine, Zhengzhou, 451200, China; ^4^ Department of Oncology, The Second Affiliated Hospital of Chongqing Medical University, Chongqing, 400010, China, cqmu.edu.cn

**Keywords:** asthma, biomarkers, Mendelian randomization, transcriptome

## Abstract

Asthma constitutes a widespread chronic respiratory disease with multifactorial origins, in which definitive genetic components remain incompletely understood. This investigation synthesized transcriptomic data and Mendelian randomization (MR) to delineate causative genes contributing to asthma predisposition. Evaluation of the GSE43696 cohort disclosed 267 genes displaying expression alterations between healthy controls (*n* = 20) and asthmatic cases (*n* = 88). MR methods discerned seven causal genes, consisting of six protective determinants (GPRIN3, SHISA2, DUSP5, FCER1A, USP36, and LGALS2; OR < 1) and one risk‐associated determinant (CXCL6; OR > 1). Independent validation with the GSE63142 series verified pronounced suppression of five genes in asthma specimens. Functional enrichment studies connected these genes to immune‐related cascades, including IL‐17/NF‐κB signal transduction and Th17 cell differentiation, alongside metabolic activities. Single‐cell transcriptomic assessment of 32,809 cells exposed compartmentalized expression profiles: FCER1A and LGALS2 predominated in dendritic cells, DUSP5 in goblet cells, SHISA2 in ionocytes, and USP36 in T cells. Immune cell infiltration evaluation demonstrated shifted distributions of mast cells, eosinophils, and monocytes, exhibiting substantial linkages with immune mediators such as CCR5 and CXCL10. Transcription factor exploration recognized cisbp__M0561 as the predominant regulatory element. Connectivity Map–based interrogation nominated KI‐8751, verrucarin‐A, and homoharringtonine as plausible therapeutic candidates. This consolidated multiomics framework elucidates previously uncharacterized pathological processes and prospective treatment avenues for asthma.

## 1. Introduction

The chronic inflammatory disease asthma involves chronic airway inflammation, hyperresponsive airways, remodeling, and structural changes in the airways [1]. The epidemiological characteristics of asthma demonstrate its global nature and impact as a complex chronic disease that requires comprehensive multidisciplinary intervention and management strategies to control and treat it [2, 3]. Scientific and reasonable treatment strategies and lifestyle management can effectively control this condition, reduce the frequency of attacks, and thereby improve quality of life [4–6]. Therefore, identifying causal genes and reliable biomarkers is crucial for elucidating pathogenesis, developing targeted therapies, and improving prognosis.

Mendelian randomization (MR) employs genetic variants—most commonly single‐nucleotide polymorphisms (SNPs)—as instrumental variables (IVs) to infer causal relationships between exposures and clinically important outcomes, operating under the assumptions of Mendelian inheritance [7]. This technique offers the advantages of mitigating confounding bias attributable to reverse causality and reducing estimation bias arising from measurement errors [8, 9].

This study aimed to identify genes causally associated with asthma risk by integrating differential expression analysis with MR. Through external validation, functional enrichment, and immune profiling, we sought to reveal the mechanistic roles of key genes and provide insights for targeted intervention.

## 2. Materials

### 2.1. Data Download

Transcriptomic data were obtained from the Gene Expression Omnibus (GEO) [10]. The discovery dataset GSE43696 (platform GPL6480) included transcriptomic profiles from 108 samples (20 healthy controls and 88 asthma patients). An independent validation dataset, GSE63142 (GPL6480), comprised 155 samples (27 controls and 128 asthma patients). Single‐cell RNA sequencing (scRNA‐seq) data were sourced from GSE164015, encompassing full transcriptomes from 8 individual airway samples.

To perform MR analysis, expression quantitative trait loci (eQTL) data for IV selection were obtained from the eQTLGen consortium (https://www.eqtlgen.org) [11]. This resource provides cis‐eQTL summary statistics derived from blood samples of 31,684 individuals, predominantly of European ancestry.

Summary‐level genome‐wide association study (GWAS) data for the asthma outcome were sourced from the FinnGen biobank (release R12, finngen_R12_J10_ASTHMA_EXMORE). This cohort includes 319,739 individuals of Finnish ancestry (59,100 asthma cases and 260,639 controls), with disease classification based on ICD‐10 codes.

A critical prerequisite for two‐sample MR is the use of independent, nonoverlapping cohorts for exposure and outcome data. Our analysis satisfies this condition, as the eQTLGen (exposure) and FinnGen (outcome) datasets originate from distinct populations. Their shared European ancestry ensures genetic homogeneity, minimizing bias from population stratification and strengthening the validity of our causal inferences.

### 2.2. Differentially Expressed Gene (DEG) Analysis

Differential gene expression between asthma patients and healthy controls in the primary GSE43696 dataset was identified using the limma package (v3.58.1) in R. Genes meeting the thresholds of an absolute log2 fold change (|logFC|) > 0.585 and a Benjamini–Hochberg adjusted *p* value < 0.05 were defined as DEGs. The results were visualized using a volcano plot.

### 2.3. MR Analysis

We employed a two‐sample MR framework using the TwoSampleMR R package to infer causal relationships between gene expression (exposure) and asthma risk (outcome). The analysis strictly adhered to the three core MR assumptions, implemented through the following pipeline:

1. IV selection: For each candidate DEG, SNPs significantly associated with its expression level (cis‐eQTLs) at a genome‐wide threshold of (*p* < 1 × 10^−5^) in the eQTLGen data were selected as potential IVs.
2. IV clustering and strength filtering: To ensure independence among IVs, linkage disequilibrium (LD) clumping was performed ((*r* ^2^ < 0.001), window = 10,000 kb). To mitigate weak instrument bias, only SNPs with an *F*‐statistic > 10 were retained.
3. Data harmonization: The selected IVs were harmonized with the outcome GWAS summary statistics from FinnGen to align effect alleles and remove palindromic or incompatible SNPs.
4. MR estimation and robustness assessment: The primary causal estimate for each gene was derived using the inverse‐variance weighted (IVW) method. To ensure robustness and control for horizontal pleiotropy, we supplemented this with three additional methods: MR‐Egger, weighted median, and weighted mode. A causal relationship was considered statistically significant only if at least two methods yielded concordant effect directions with a nominal (*p* < 0.05).
5. Sensitivity analyses: We performed comprehensive sensitivity testing: (i) Cochran’s *Q* test to assess heterogeneity among IVs; (ii) the MR‐Egger intercept test to evaluate directional pleiotropy; (iii) the MR‐PRESSO global test to detect and correct for outlier SNPs; and (iv) leave‐one‐out analysis to verify that results were not driven by any single SNP.
6. Multiple testing correction: We employed a hierarchical correction strategy. A Bonferroni‐corrected threshold ((*p* < 0.05/*n*), where *n* is the number of candidate genes tested) was applied at the gene discovery level. The requirement for concordance across multiple MR methods and passage of sensitivity tests provided an additional layer of false discovery rate control.

### 2.4. Heterogeneity Test

To assess potential statistical heterogeneity among the analyzed SNPs, we applied a heterogeneity test in the MR framework. The *Q* statistic was calculated as the weighted sum of squared differences between individual SNP effect estimates and the pooled effect, divided by their variances. This statistic follows a *χ* ^2^ distribution with degrees of freedom corresponding to the number of instrumental variants minus one. A resulting *p* value greater than 0.05 implies a lack of significant heterogeneity, supporting consistency across genetic instruments in their estimated effects on disease risk.

### 2.5. Sensitivity Analysis

To evaluate the influence of individual genetic variants on asthma, a leave‐one‐out sensitivity analysis was implemented within the MR framework. This procedure iteratively removes each SNP and recalculates the pooled effect size for the remaining variants, facilitating identification and exclusion of SNPs that disproportionately influence the overall causal estimate. The exclusion of each SNP produces an updated point estimate along with its corresponding 95% confidence interval, enabling evaluation of the variant’s specific contribution and the stability of the aggregated result. The resulting estimates are summarized graphically, displaying values obtained after each sequential exclusion alongside the combined estimate incorporating all SNPs. Comparative assessment of these values visualizes the effect of excluding each individual SNP on the overall association and validates the robustness of the analytical approach.

### 2.6. Functional Enrichment Analysis

Gene set enrichment analysis (GSEA) was conducted using the clusterProfiler R package to identify signaling pathways enriched in samples with high versus low expression of key genes. Gene set variation analysis (GSVA) was performed using the GSVA package to calculate sample‐wise pathway activity scores. Gene sets were sourced from the Hallmark and Canonical Pathways collections in the Molecular Signatures Database (MSigDB). Pathways with an adjusted *p* value (FDR) < 0.05 were deemed significant.

### 2.7. Immune Infiltration

The relative abundances of 22 human immune cell subtypes within the bulk tissue transcriptomic samples were estimated using the CIBERSORT algorithm with its default LM22 signature matrix (547 genes). This deconvolution approach allowed for correlation analysis between key gene expression and estimated immune cell proportions.

### 2.8. Transcription Factor Regulatory Network

To identify potential upstream transcriptional regulators of the key gene set, we performed motif enrichment analysis using the RcisTarget R package. This method evaluates the overrepresentation of conserved DNA binding motifs within the regulatory regions (promoters) of the input genes, reporting normalized enrichment scores (NESs) for each motif.

### 2.9. Single‐Cell Sequencing Data Quality Control and Data Normalization

The scRNA‐seq data (GSE164015) were processed using the Seurat toolkit (v4.3.0). Quality control involved filtering out low‐quality cells (expressing < 200 genes or with high mitochondrial RNA content) and doublets (identified using DoubletFinder). Data were normalized, scaled to regress out unwanted sources of variation (e.g., mitochondrial gene expression), and integrated across samples using Harmony to correct for batch effects. Cell clusters were identified via principal component analysis (PCA) followed by graph‐based clustering in a shared nearest neighbor (SNN) graph. Cell types were annotated by cross‐referencing the expression of canonical marker genes with the CellMarker database and automated annotation via the SingleR package. The expression of key genes was visualized on uniform manifold approximation and projection (UMAP) embeddings.

### 2.10. Statistical Analysis

MR analysis is based on three core assumptions: relevance (genetic instruments must be robustly associated with the exposure), independence (instruments must not be associated with confounders), and exclusion restriction (instruments affect the outcome only through the exposure; violations indicate pleiotropy). All analyses were conducted in R software (Version 4.3.0). A two‐sided *p* value threshold of 0.05 was used to determine statistical significance. To address multiple testing in the MR analysis, a hierarchical correction strategy was employed, as detailed in Section 2.3.

## 3. Results

### 3.1. Identification of DEGs in Asthma

The asthma‐associated dataset GSE43696 was acquired from the GEO repository, comprising a control cohort (*n* = 20) and an asthmatic cohort (*n* = 88). Differential gene expression between healthy and diseased subjects was evaluated utilizing the limma package. Genes were classified as differentially expressed based on thresholds of *p* value < 0.05 and an absolute log‐fold change greater than 0.585. In total, 267 genes displayed differential expression, of which 116 were upregulated and 151 were downregulated (Figures 1(a) and 1(b)).

FIGURE 1Integrative profiling of differential expression and Mendelian randomization. (a) Volcano plot depicting genes exhibiting expression alterations, with blue representing suppressed genes and pink signifying upregulated ones. (b) Heat map visualizing genes displaying expression changes, where blue corresponds to diminished expression and pink indicates elevated expression. (c–k) Graphical representations of hazard ratios and associated *p* values for seven causal associations identified through Mendelian randomization.

**Figure:**
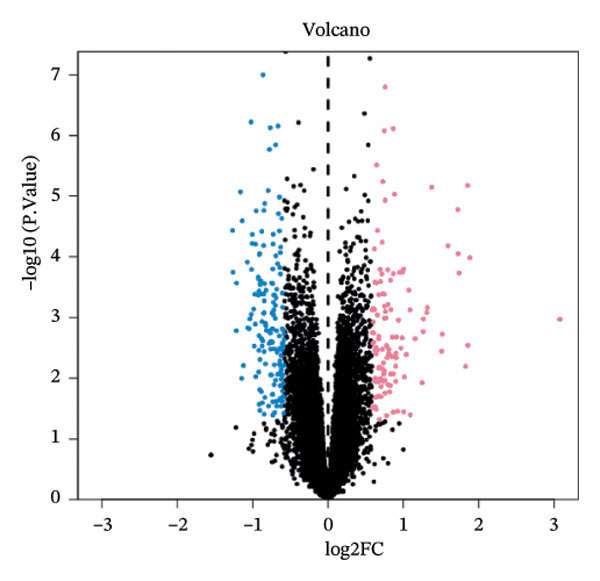
(a)

**Figure:**
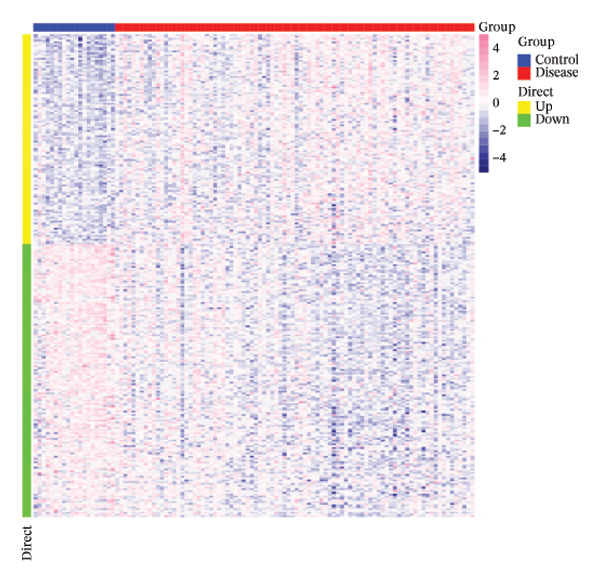
(b)

**Figure:**
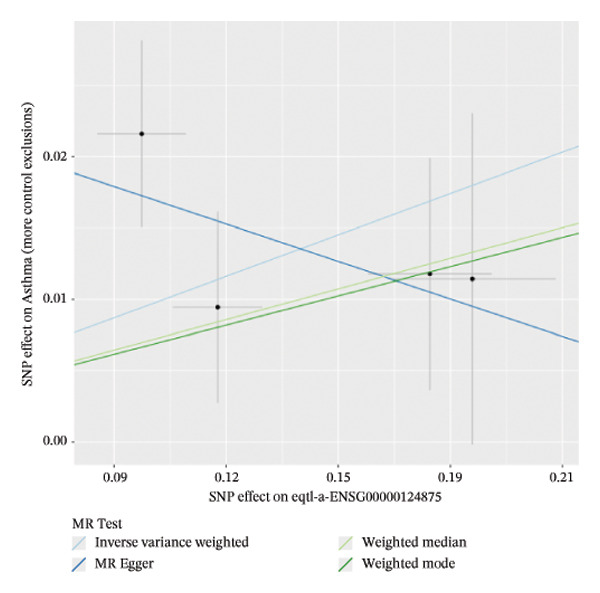
(c)

**Figure:**
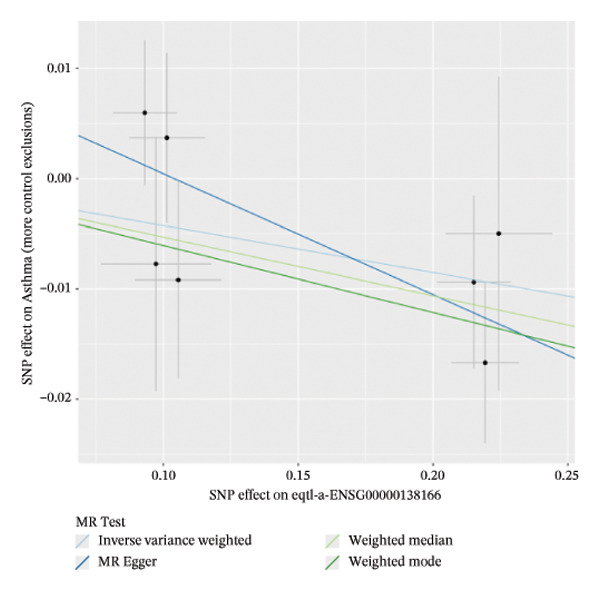
(d)

**Figure:**
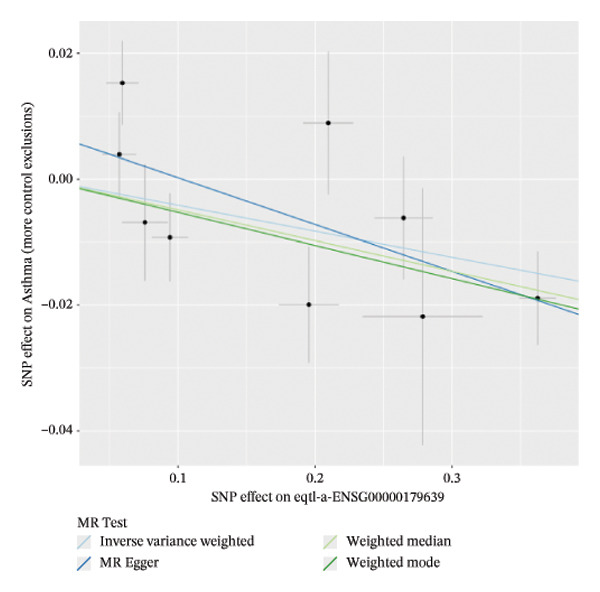
(e)

**Figure:**
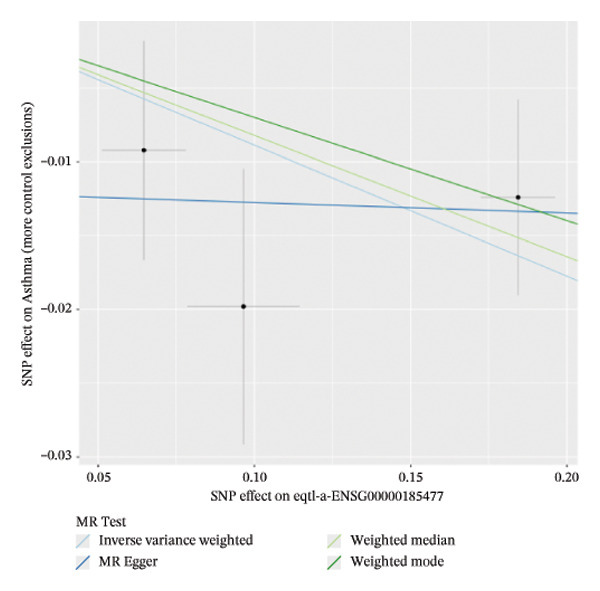
(f)

**Figure:**
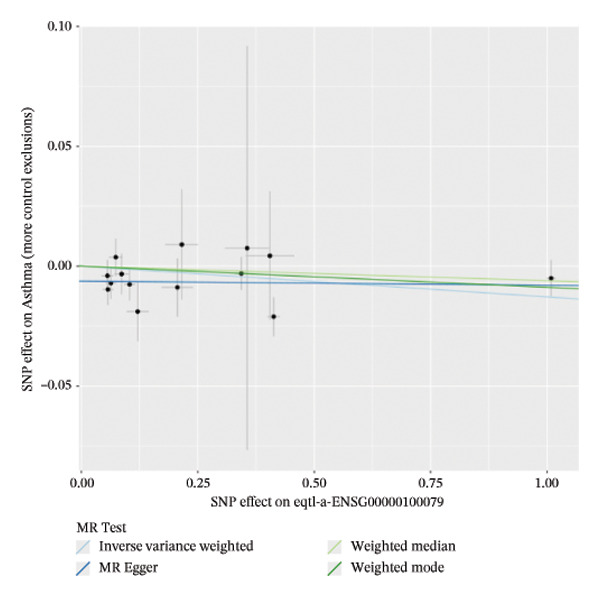
(g)

**Figure:**
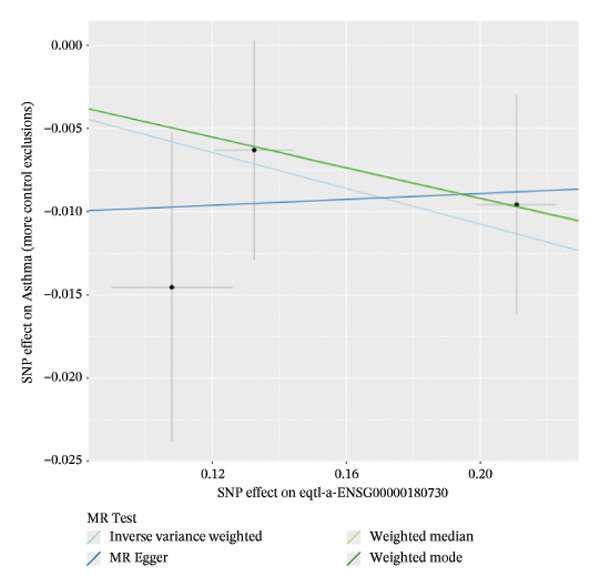
(h)

**Figure:**
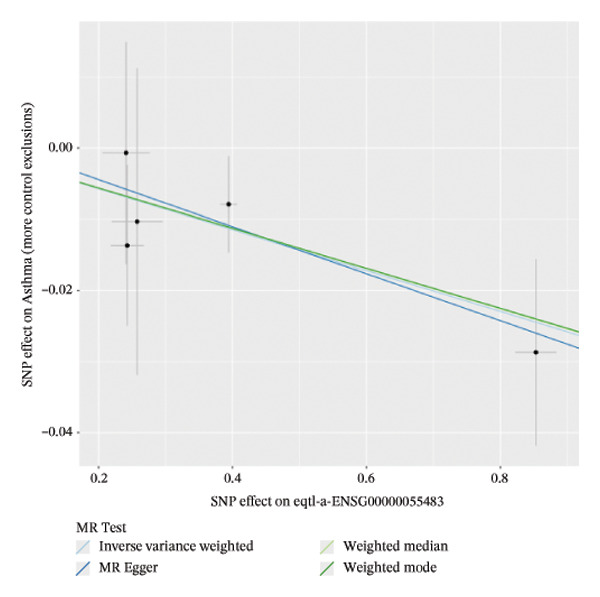
(i)

**Figure:**
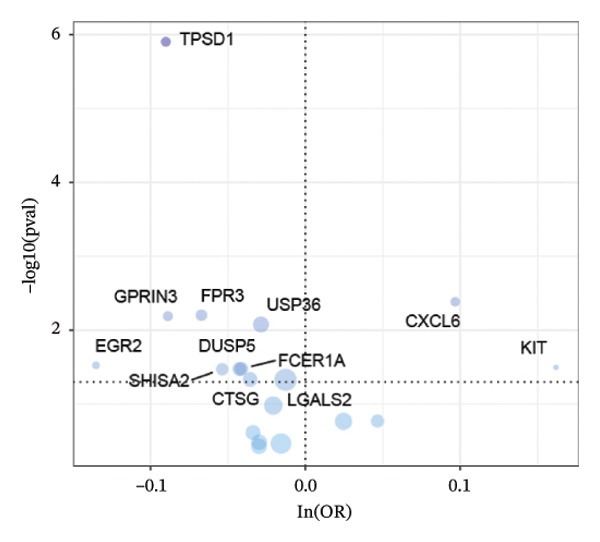
(j)

**Figure:**
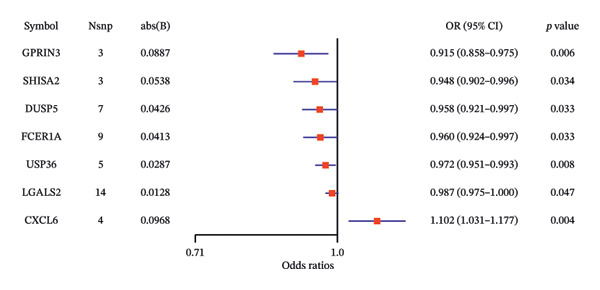
(k)

### 3.2. MR Identifies Causal Asthma‐Related Genes

The DEGs identified in the previous step underwent MR analysis. Causal relationships between 19 eQTLs and outcomes were extracted using extract_instruments and extract_outcome_data functions. Subsequent MR analysis pinpointed seven gene–outcome pairs exhibiting significant causal relationships with eQTL‐positive outcomes (Figures 1(c), 1(d), 1(e), 1(f), 1(g), 1(h), 1(i), 1(j), and 1(k); IVW *p* < 0.05). Results indicated that six genes—GPRIN3 (OR = 0.915; 95% CI: 0.858–0.975; *p* = 0.006), SHISA2 (OR = 0.948; 95% CI: 0.902–0.996; *p* = 0.034), DUSP5 (OR = 0.958; 95% CI: 0.921–0.997; *p* = 0.034), FCER1A (OR = 0.960; 95% CI: 0.924–0.997; *p* = 0.033), USP36 (OR = 0.972; 95% CI: 0.951–0.993; *p* = 0.008), and LGALS2 (OR = 0.987; 95% CI: 0.975–0.999; *p* = 0.047)—were associated with reduced asthma risk (OR < 1). In contrast, CXCL6 (OR = 1.102; 95% CI: 1.031–1.177; *p* = 0.004) was linked to elevated asthma risk (OR > 1) (Figures 1(c), 1(d), 1(e), 1(f), 1(g), 1(h), and 1(i)). Heterogeneity tests confirmed that all seven genes met assumptions for MR analysis. Sensitivity analyses, including leave‐one‐out validation, demonstrated that excluding any single SNP did not significantly alter effect estimates, supporting the robustness of the inferred causal relationships (Figures 2(a), 2(b), 2(c), 2(d), 2(e), 2(f), and 2(g)).

FIGURE 2Leave‐one‐out sensitivity analysis and intersection of DEGs with risk‐associated genes. (a–g) Leave‐one‐out sensitivity analysis results presented as forest plots for SNPs linked to significantly associated genes. (h) Venn diagram illustrating the overlap between downregulated differentially expressed genes and genes associated with reduced risk. (i) Venn diagram depicting the intersection of upregulated differentially expressed genes and genes correlated with elevated risk.

**Figure:**
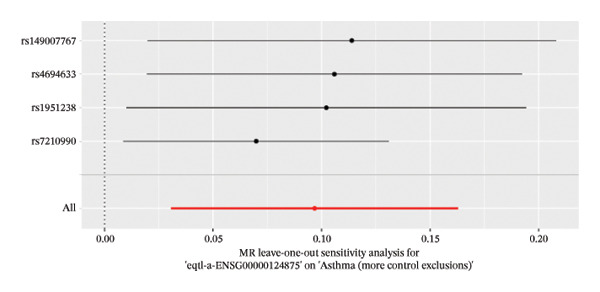
(a)

**Figure:**
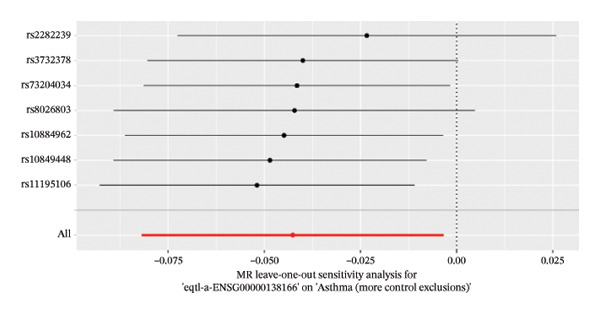
(b)

**Figure:**
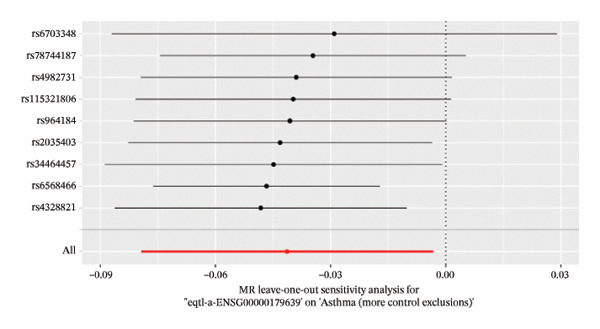
(c)

**Figure:**
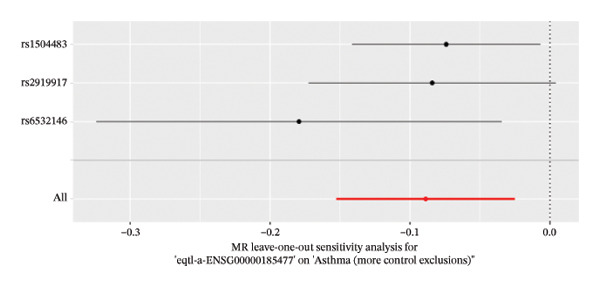
(d)

**Figure:**
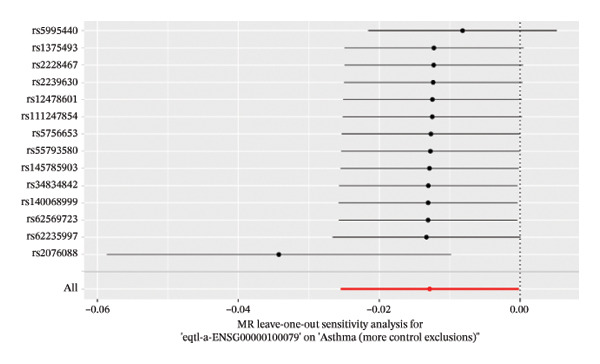
(e)

**Figure:**
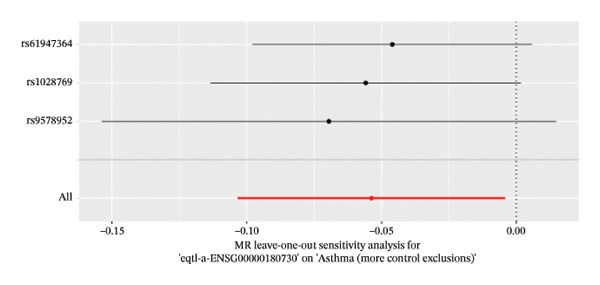
(f)

**Figure:**
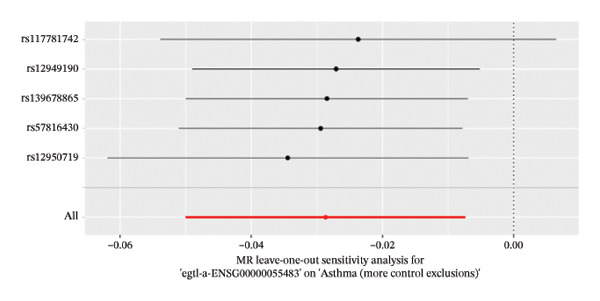
(g)

**Figure:**
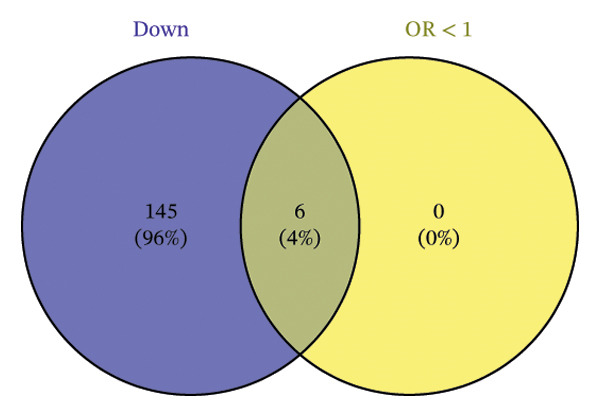
(h)

**Figure:**
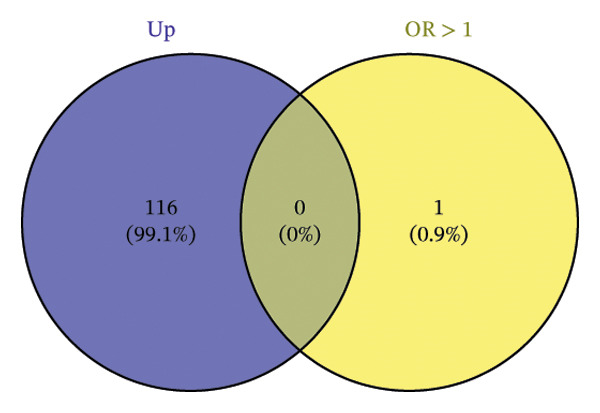
(i)

### 3.3. External Validation of MR‐Based Gene Signatures

Venn diagram analysis was employed to determine the overlap between differentially downregulated genes and those linked to decreased asthma susceptibility (OR < 1) derived from the preceding MR. Analyses identified six common genes—GPRIN3, SHISA2, DUSP5, FCER1A, USP36, and LGALS2 as depicted in Figure 2(h). Intersection results from upregulated DEGs and high‐risk associated genes (OR > 1) showed no overlapping loci (Figure 2(i)).

For further confirmation, external validation was conducted using the GSE63142 dataset retrieved from the GEO repository. Differential expression analysis between control and asthmatic cohorts unveiled notable expression disparities for five genes (DUSP5, FCER1A, LGALS2, SHISA2, and USP36) with reduced expression in disease subjects relative to healthy controls (Figure 3(a)). These observations align with the MR outcomes, thereby demonstrating that diminished expression of these genes correlates with lower asthma risk and supports the validity of our analytical approach.

FIGURE 3Validation of key gene expression and correlation analysis. (a, b) Expression box plots (a) and heat maps (b) of DUSP5, FCER1A, LGALS2, SHISA2, and USP36 in asthma and controls in the GSE63142 dataset. (c) Interaction network diagram of 5 key genes. ns, *p* > 0.05, ^∗^ *p* < 0.05, ^∗∗^ *p* < 0.01, and ^∗∗∗^ *p* < 0.001.

**Figure:**
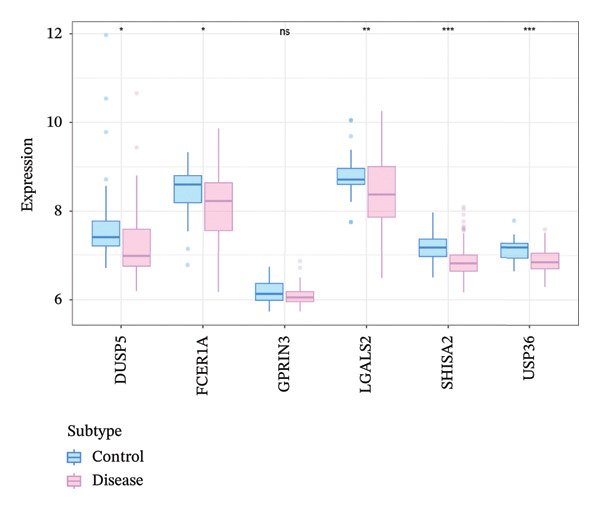
(a)

**Figure:**
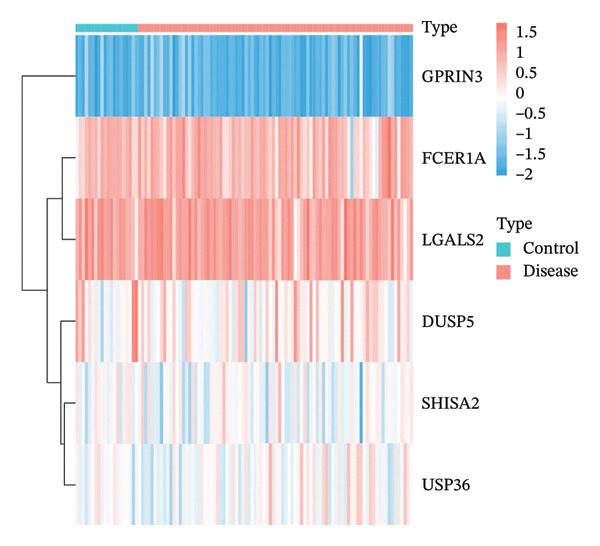
(b)

**Figure:**
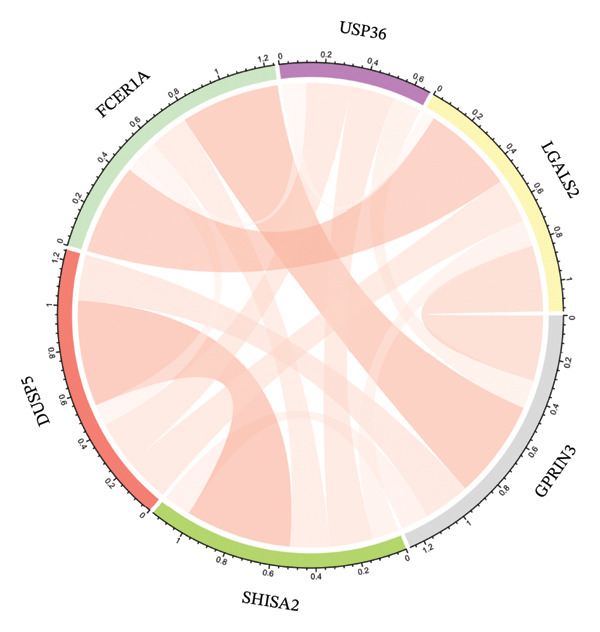
(c)

### 3.4. Functional Enrichment and Pathway Analysis

Next, we explored the interaction networks of the five key genes through correlation analysis, displaying them as circle plots and heat maps (Figures 3(b) and 3(c)). We also investigated the specific signaling pathways involved in these key genes to explore the potential molecular mechanisms by which they influence disease progression. GSEA results showed that DUSP5 was enriched in signaling pathways including the IL‐17, the B cell receptor, and the NF‐κB signaling pathway (Figure 4(a)). FCER1A was enriched in signaling pathways including the Th17 cell differentiation pathway, the Th1 and Th2 cell differentiation pathway, and the chemokine signaling pathway (Figure 4(b)). LGALS2 was enriched in signaling pathways including the Th17 cell differentiation, the chemokine, and the NF‐kappa B signaling pathway (Figure 4(c)). SHISA2 was enriched in signaling pathways including the Th1 and Th2 cell differentiation pathway, the Th17 cell differentiation, and the mineral absorption (Figure 4(d)). USP36 was enriched in signaling pathways such as biosynthesis of nucleotide sugars, steroid hormone biosynthesis, and mucin type O‐glycan biosynthesis (Figure 4(e)).

FIGURE 4Signaling pathway analyses of key genes. (a–e) Enriched KEGG pathways associated with key genes, depicting pathway activity and participant genes. (f–j) GSVA enrichment profiles for key genes, where blue denotes pathways linked to highly expressed genes and green indicates those associated with lowly expressed genes, based on the Hallmark gene set as reference.

**Figure:**
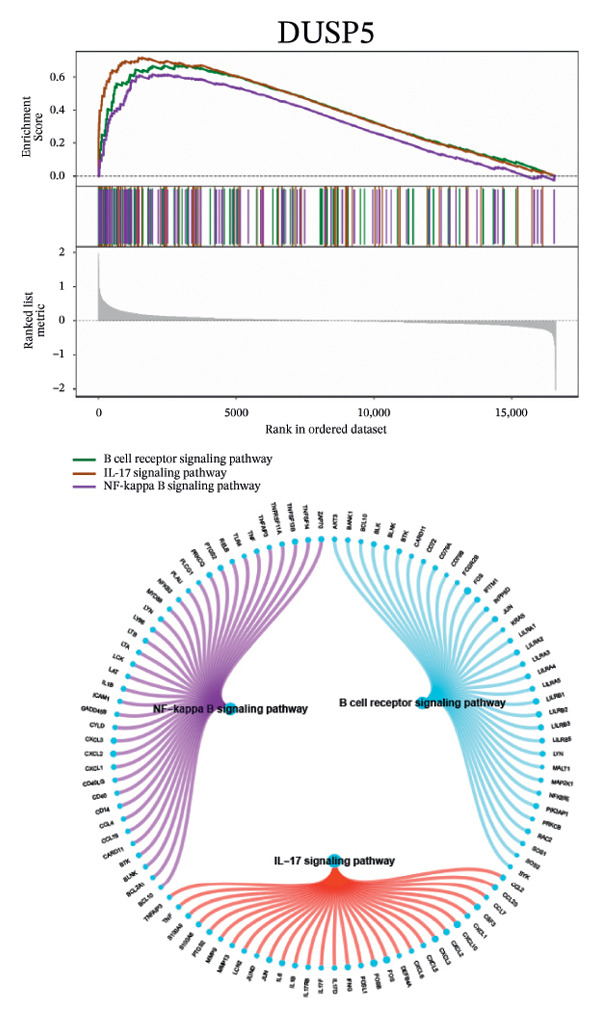
(a)

**Figure:**
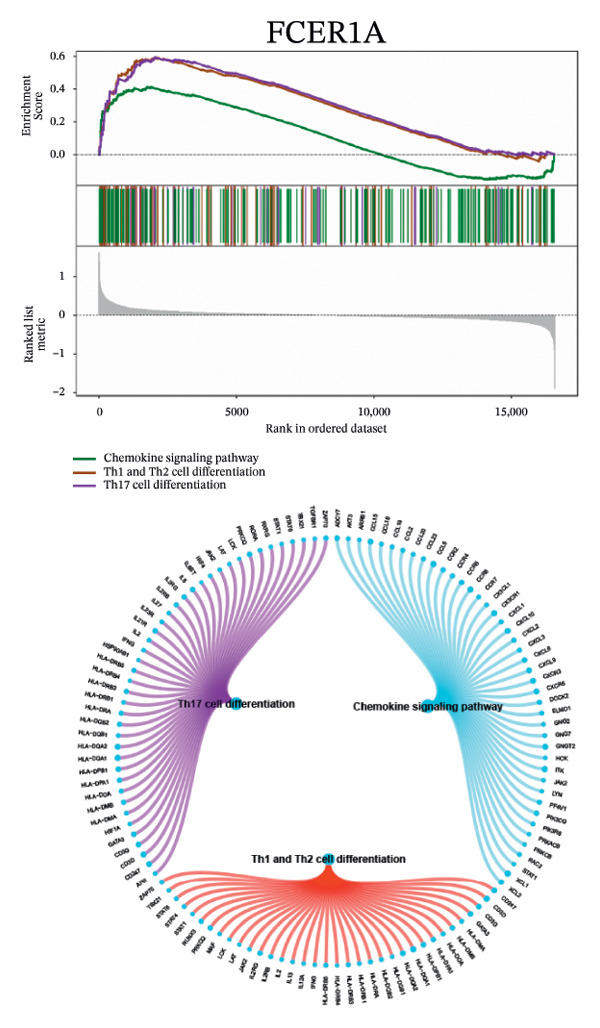
(b)

**Figure:**
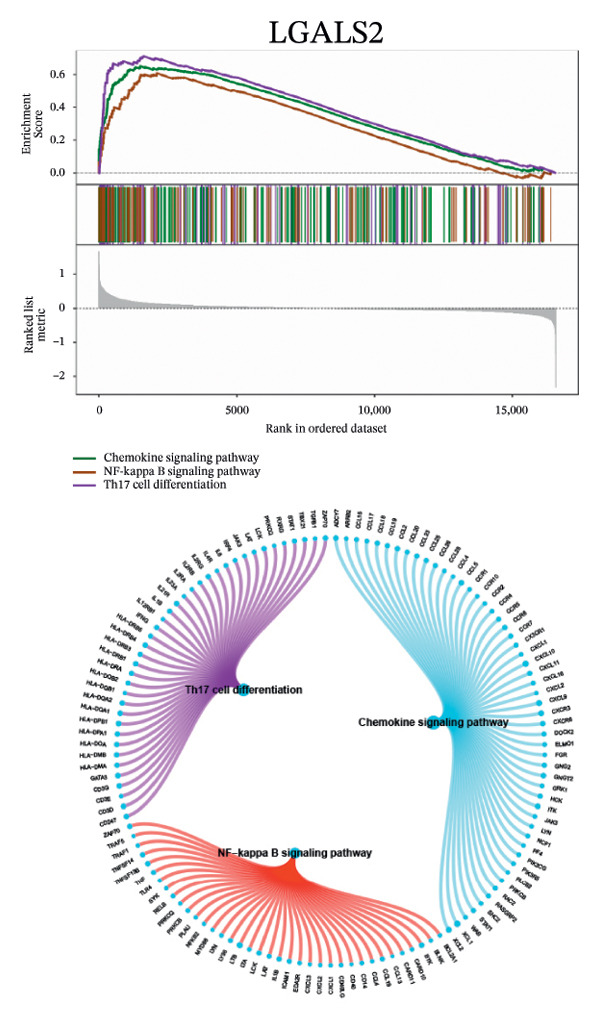
(c)

**Figure:**
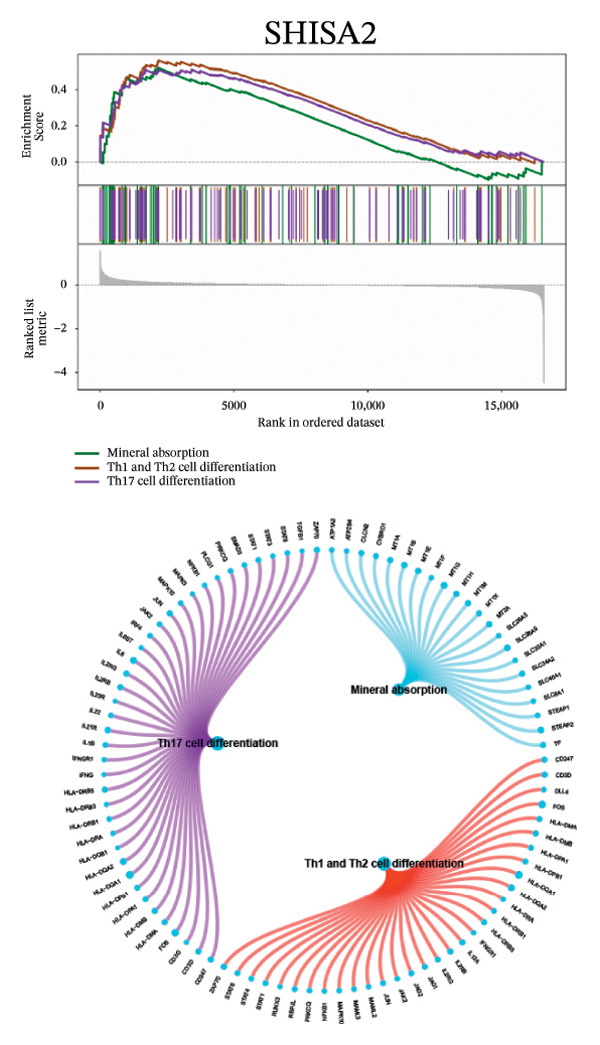
(d)

**Figure:**
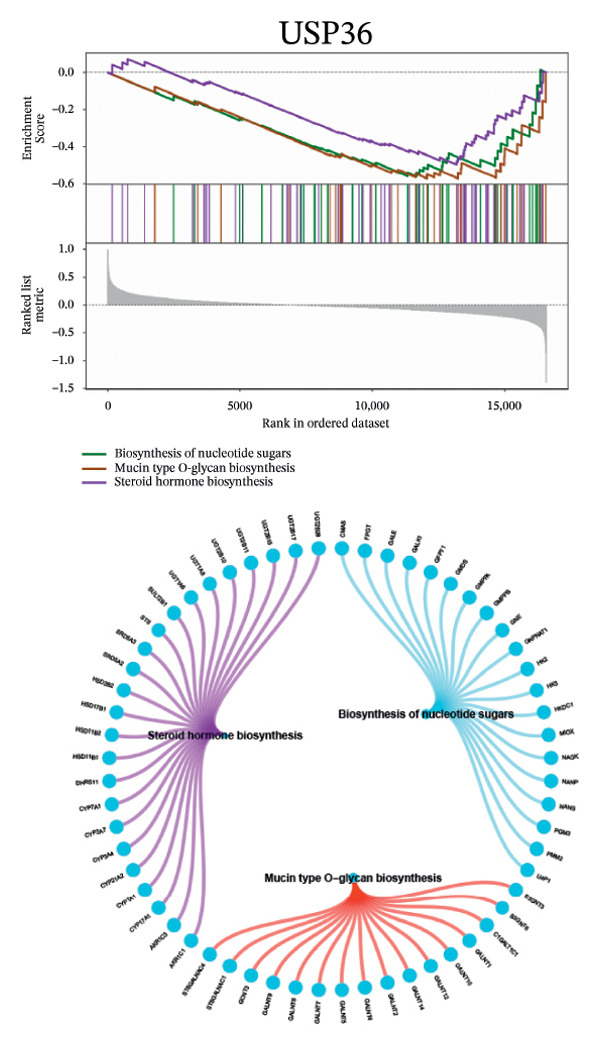
(e)

**Figure:**
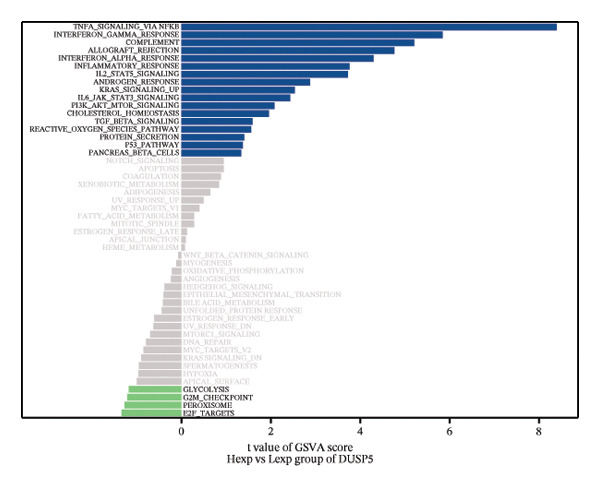
(f)

**Figure:**
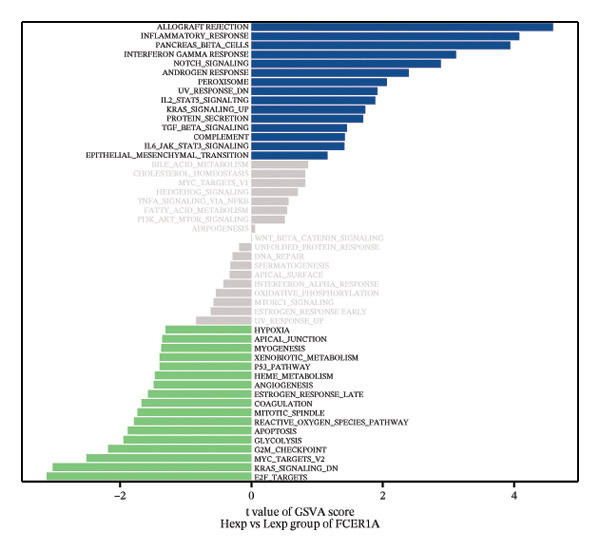
(g)

**Figure:**
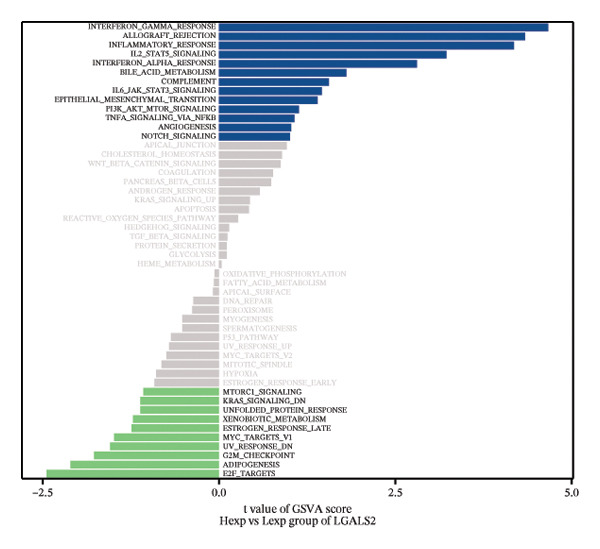
(h)

**Figure:**
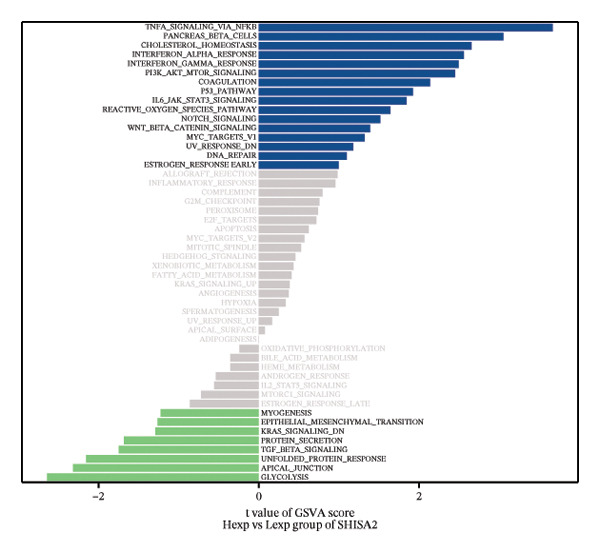
(i)

**Figure:**
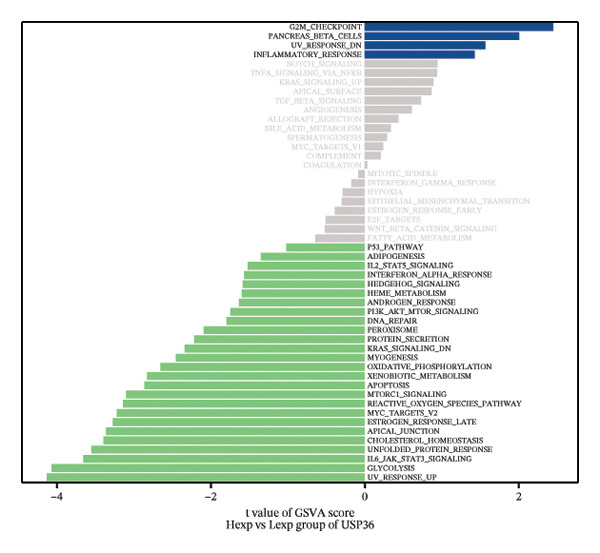
(j)

GSVA analysis showed that DUSP5 was enriched in signaling pathways such as TNFA_SIGNALING_VIA_NFKB and INTERFERON_GAMMA_RESPONSE (Figure 4(f)). FCER1A was enriched in signaling pathways such as ALLOGRAFT_REJECTION and INFLAMMATORY_RESPONSE (Figure 4(g)). LGALS2 was enriched in signaling pathways such as INTERFERON_GAMMA_RESPONSE and ALLOGRAFT_REJECTION (Figure 4(h)). SHISA2 was enriched in signaling pathways such as TNFA_SIGNALING_VIA_NFKB and PANCREAS_BETA_CELLS (Figure 4(i)). USP36 is enriched in signaling pathways such as G2M_CHECKPOINT and PANCREAS_BETA_CELLS (Figure 4(j)). This suggests that key genes may influence disease progression through these pathways.

### 3.5. Immune Infiltration and Microenvironment Analysis

The tumor microenvironment plays a pivotal role in shaping disease diagnosis, patient survival, and therapeutic outcomes, as it integrates fibroblasts, immune populations, extracellular matrix components, various growth and inflammatory mediators, and specific biophysical characteristics. Our examination of immune infiltration profiles and their connections to different immune subtypes utilized several computational strategies (Figures 5(a) and 5(b)). Evaluations comparing disease and control groups identified elevated levels of resting mast cells among pathological cases, accompanied by substantial declines in memory B cells, eosinophils, monocytes, and naïve T lymphocytes (Figure 5(c)). Additional assessments of interactions between pivotal genes and immune components indicated that DUSP5 levels were positively related to activated CD4 memory T cells and follicular helper T cells, while being inversely associated with resting CD4 memory T cells and resting mast cells. FCER1A expression displayed positive relationships with M2 macrophages and resting dendritic cells, yet showed negative correlations with M1 macrophages and neutrophils. For LGALS2, increased expression was positively linked to activated NK cells and resting dendritic cells, but showed inverse relationships with resting CD4 memory T cells and resting NK cells. SHISA2 levels were positively aligned with follicular helper T cells and monocytes, but inversely connected to plasma cells and resting mast cells. USP36 transcriptional activity correlated positively with memory B cells and activated CD4 memory T cells, while being negatively associated with plasma cells and resting CD4 memory T cells (Figure 5(d)).

FIGURE 5Immune cell infiltration assessment. (a) Proportional distributions of immune cell subpopulations between control and asthmatic cohorts. (b) Intercellular correlations among immune subsets, where blue denotes inverse relationships and red signifies direct associations. (c) Disparities in immune cell infiltration levels between healthy and diseased groups, where blue signifies control individuals and pink indicates affected subjects. (d) Relationships between pivotal genes and immune cell infiltration content. ns, *p* > 0.05, ^∗^ *p* < 0.05, and ^∗∗^ *p* < 0.01.

**Figure:**
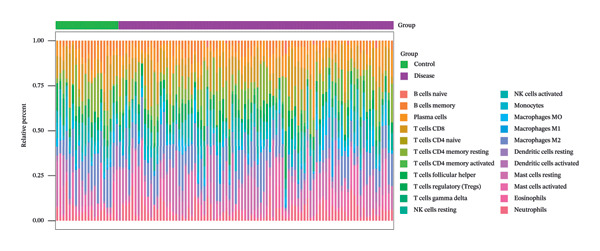
(a)

**Figure:**
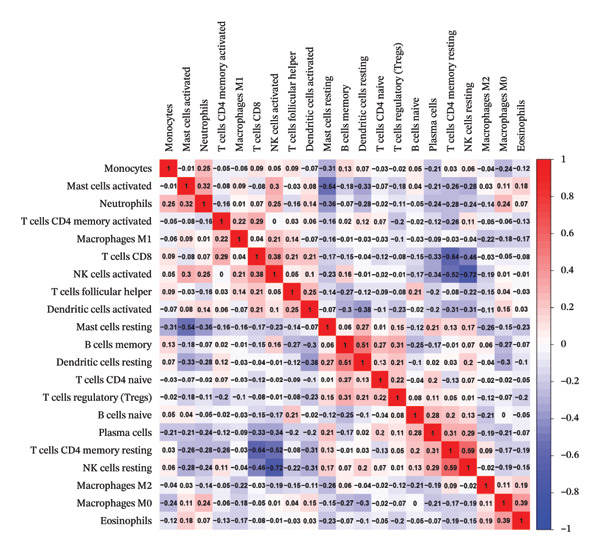
(b)

**Figure:**
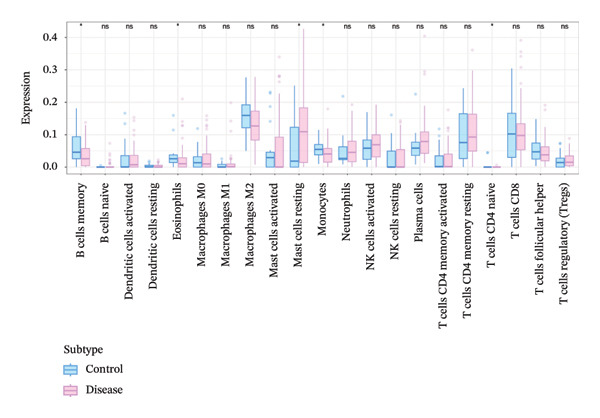
(c)

**Figure:**
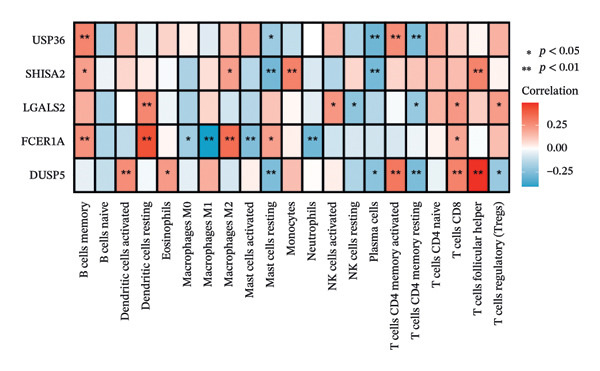
(d)

### 3.6. Immune Modulator and Transcriptional Regulation

Furthermore, we examined the relationships between key genes and diverse immune components—such as immunosuppressive factors, immunostimulatory molecules, chemokines, and receptors—which revealed strong associations between these genes and immune infiltration levels, underscoring their functional significance within the immune microenvironment (Figures 6(a), 6(b), 6(c), 6(d), and 6(e)). Notably, DUSP5, FCER1A, and LGALS2 exhibited highly consistent and symmetric correlation patterns across multiple immune factors. For instance, all three genes displayed significant positive correlations with chemokines including XCL2, CXCL9, CXCL10, and CCL5, as well as with receptors such as CCR4, CCR5, CCR6, and CXCR6.

FIGURE 6Correlation between key genes and immune factors. (a) Associations of pivotal genes with chemokine expression profiles. (b) Relationships between central genes and immunoinhibitory factors. (c) Linkages of hub genes with immunostimulatory molecules. (d) Correlations of key genes with major histocompatibility complex components. (e) Interactions between pivotal genes and immune receptor expression.

**Figure:**
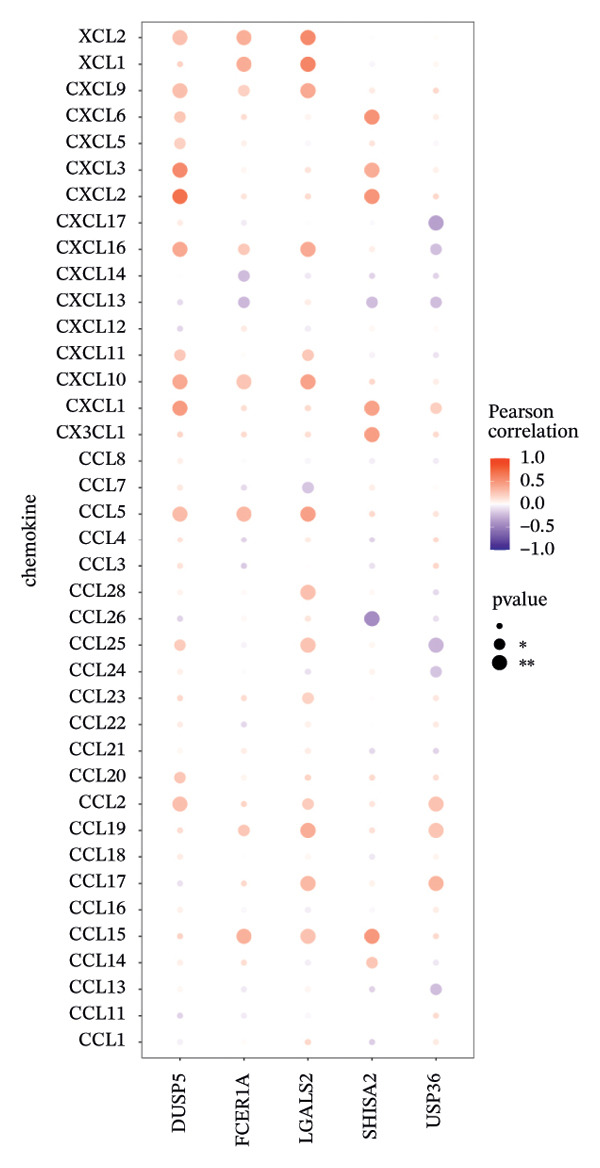
(a)

**Figure:**
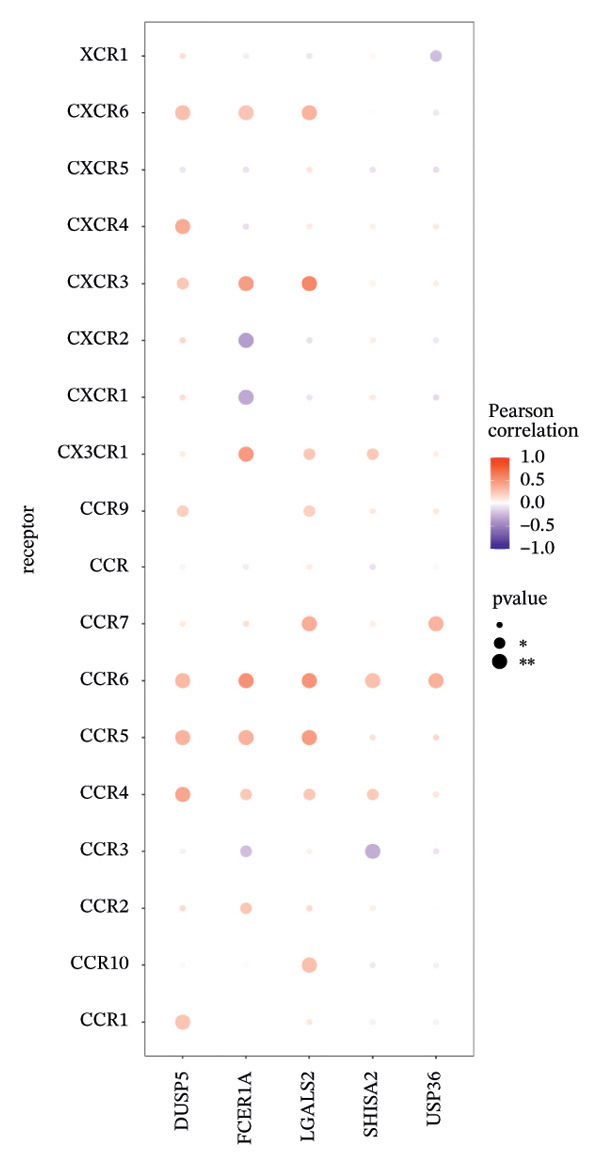
(b)

**Figure:**
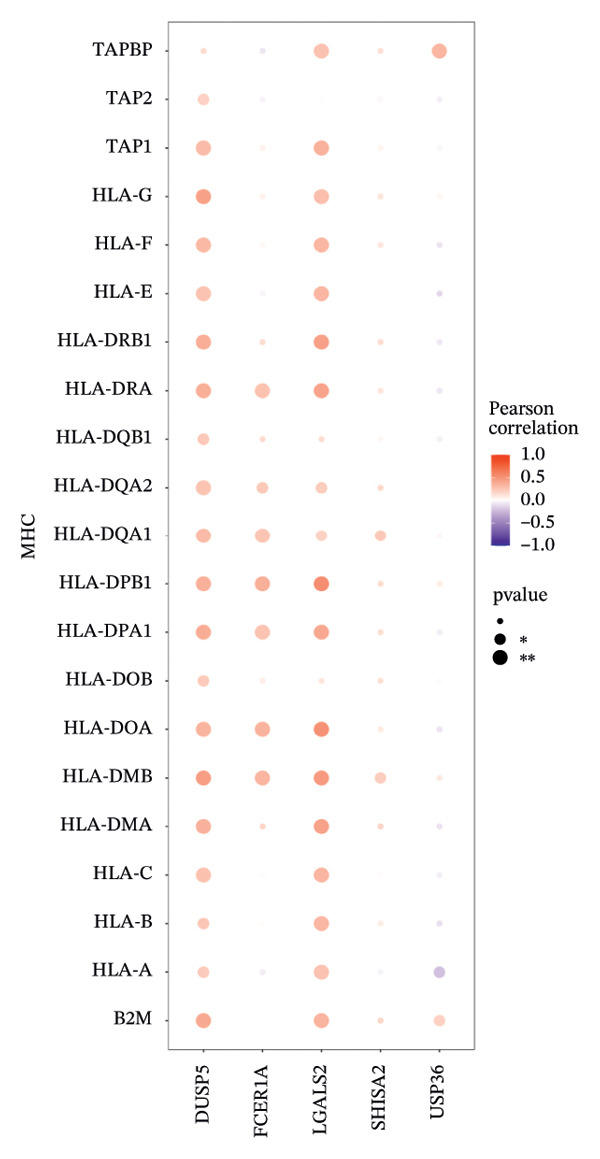
(c)

**Figure:**
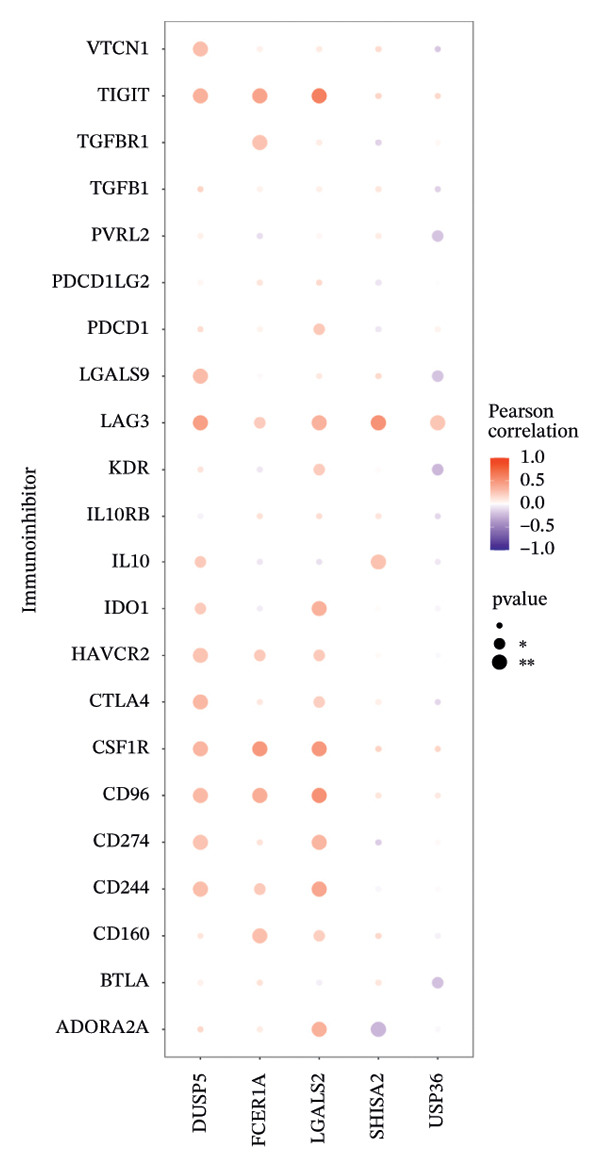
(d)

**Figure:**
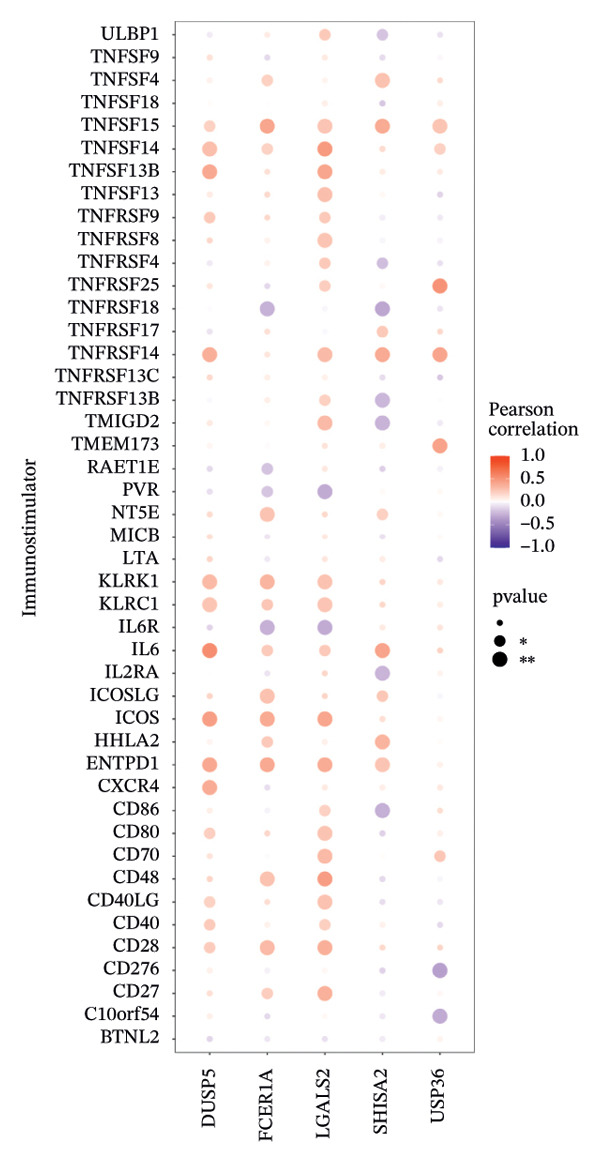
(e)

We next focused on these key genes as a target set and identified evidence of coregulation by shared transcription factors. Enrichment analysis using cumulative recovery curves highlighted cisbp__M0561 as the most significantly enriched motif (NES = 5.78). The top 15 enriched motifs and their corresponding transcription factors are presented in Figures 7(a) and 7(b).

FIGURE 7Transcriptional regulatory network of key genes. (a) Regulatory network between key genes and transcription factors. Red indicates key genes; green indicates transcription factors. (b) Display of all enriched motifs and corresponding transcription factors for key genes.

**Figure:**
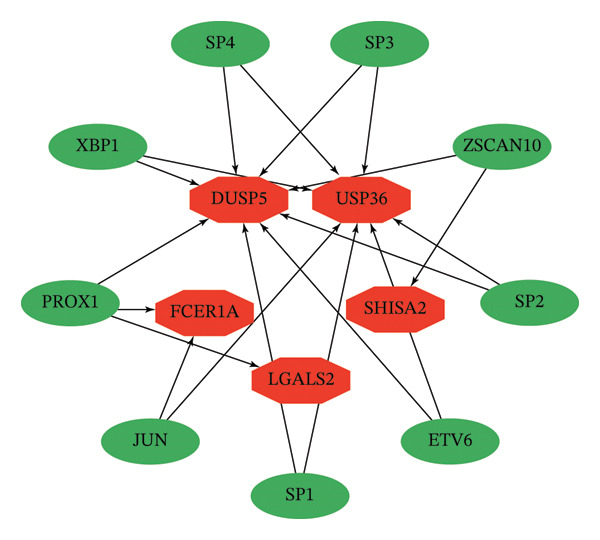
(a)

**Figure:**
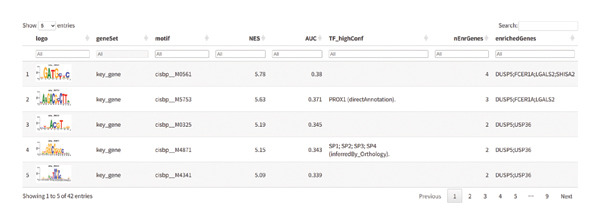
(b)

### 3.7. Single‐Cell Transcriptomic Profiling of Airway Cells

Initial data processing involved importing expression profiles through the Seurat package. Rigorous quality control was implemented across all samples to exclude outliers and low‐quality cells expressing fewer than 200 genes. Doublets were subsequently removed using the DoubletFinder algorithm, yielding a final cohort of 32,809 high‐quality cells for further investigation. Post‐filtering visualizations, including violin and scatter plots, are displayed in Figures S1A and S1B. We detected 2,000 highly variable genes (Figure S1C), and the dataset underwent normalization, integration, PCA, and Harmony‐based batch correction (Figures S1D, S1E, and S1F).

Dimensionality reduction via UMAP segregated cells into 15 distinct clusters (Figure 8(a)). Annotation using established cellular markers classified these clusters into nine cell types: ciliated cells, goblet cells, T cells, dendritic cells, basal cells, monocytes, mast cells, B cells, and ionocytes (Figure 8(b)). Bubble plots illustrate the expression distribution of canonical marker genes for each cell type (Figure 8(c)).

FIGURE 8Cell annotation and expression of key genes in single cells. (a) Cellular clustering via UMAP delineated 15 distinct subpopulations guided by principal components derived from PCA. (b) Annotation of these clusters identified nine major cell types: ciliated cells, goblet cells, T cells, dendritic cells, basal cells, monocytes, mast cells, B cells, and ionocytes. (c) Dot plot visualizing marker gene expression patterns across the nine annotated cell types. (d) Scatter plot depicting expression patterns of pivotal genes among the nine cell populations. (e) Bubble chart representing expression levels of key genes across all cell types, where blue denotes low expression and red signifies elevated expression.

**Figure:**
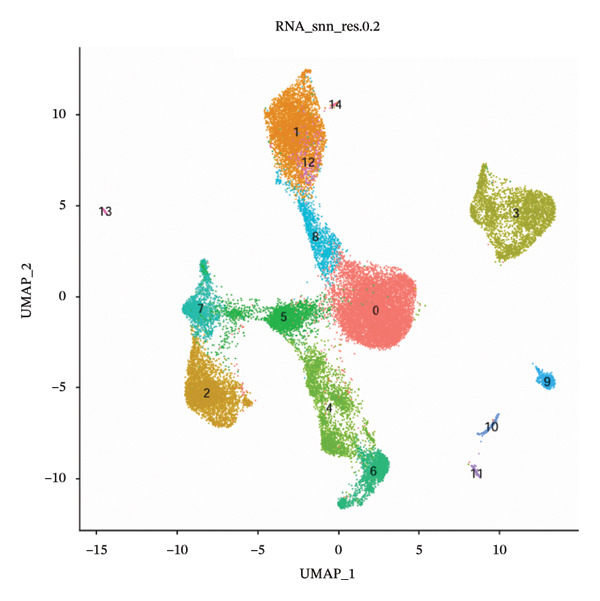
(a)

**Figure:**
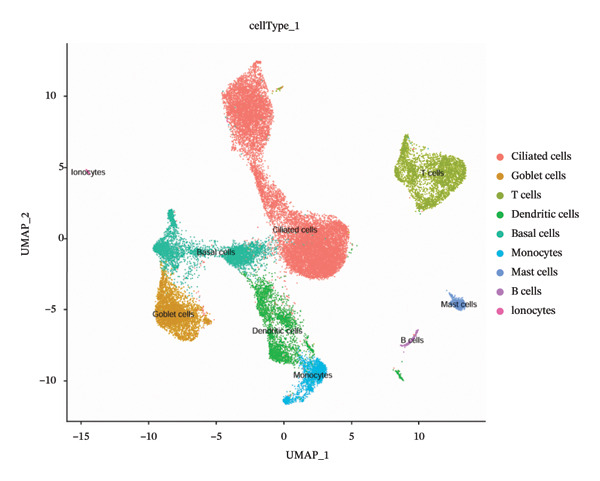
(b)

**Figure:**
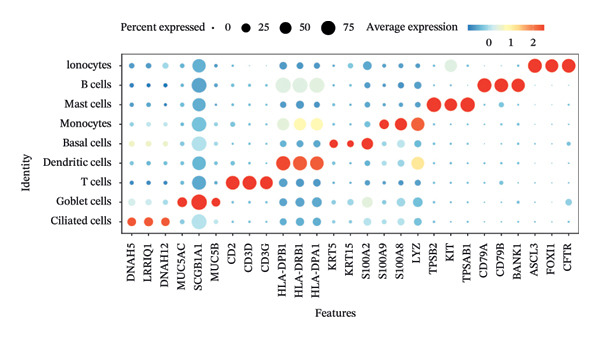
(c)

**Figure:**
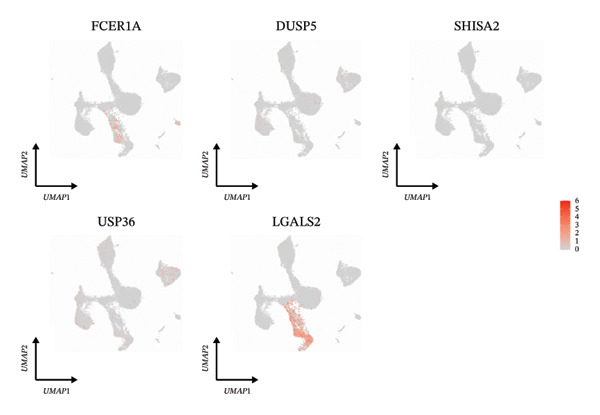
(d)

**Figure:**
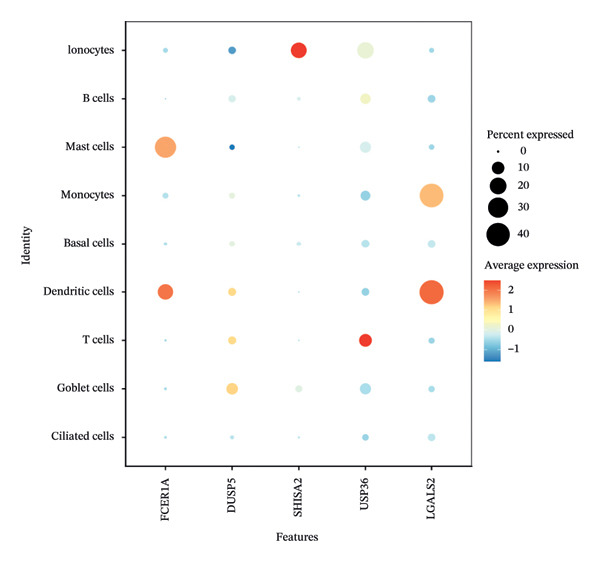
(e)

We next examined single‐cell expression patterns of key genes, visualizing their distributions with Dot plot and Feature Plot functions in Seurat. FCER1A and LGALS2 exhibited elevated expression in dendritic cells; DUSP5 was enriched in goblet cells; SHISA2 showed predominant expression in ionocytes; and USP36 was highly expressed in T cells (Figures 8(d) and 8(e)). Using AUCell, we quantified activity scores for immune‐metabolic pathways at single‐cell resolution and visualized differential gene expression via bubble plots. Results indicated strong pathway activity for DUSP5, FCER1A, LGALS2, SHISA2, and USP36 in oxidative phosphorylation, mTORC1 signaling, unfolded protein response, and MYC targets v1 (Figure 9(a)).

FIGURE 9Activity differences in immune‐metabolic pathways of key genes and drug prediction. (a) Activity variations across key genes and immune‐metabolic pathways, where blue denotes reduced activity and red represents elevated activity. (b–e) Two‐dimensional molecular structures of candidate compounds identified through Connectivity Map screening: KI‐8751, verrucarin‐A, tyrphostin‐AG‐126, and homoharringtonine.

**Figure:**
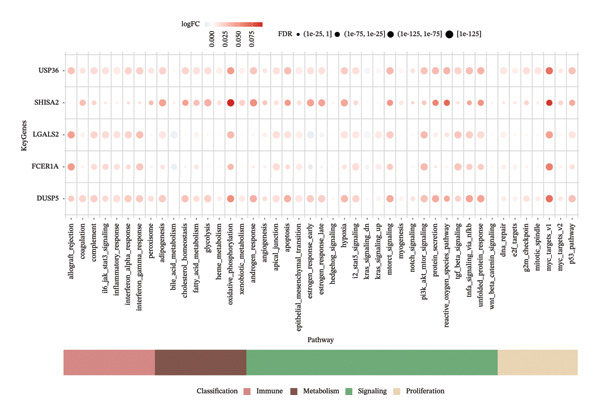
(a)

**Figure:**
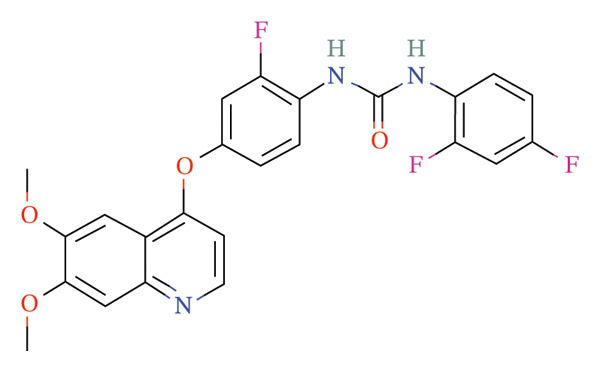
(b)

**Figure:**
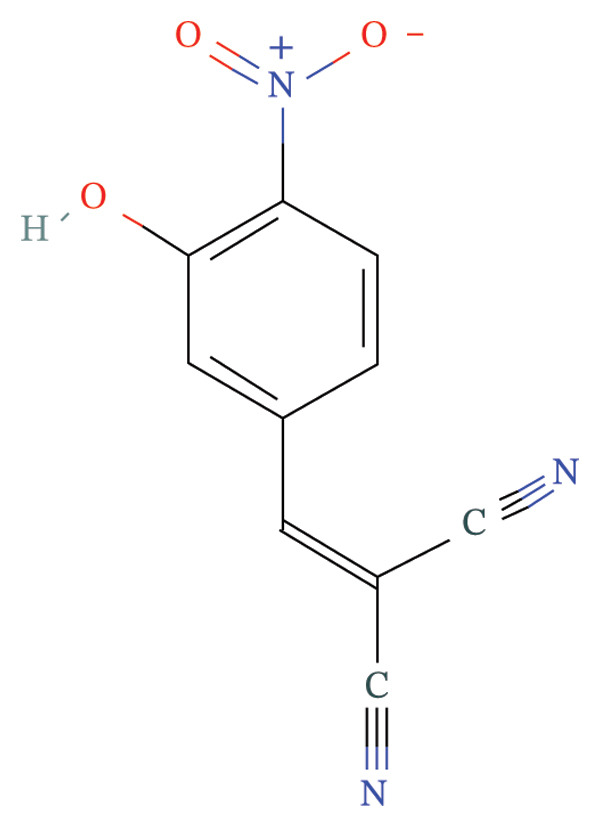
(c)

**Figure:**
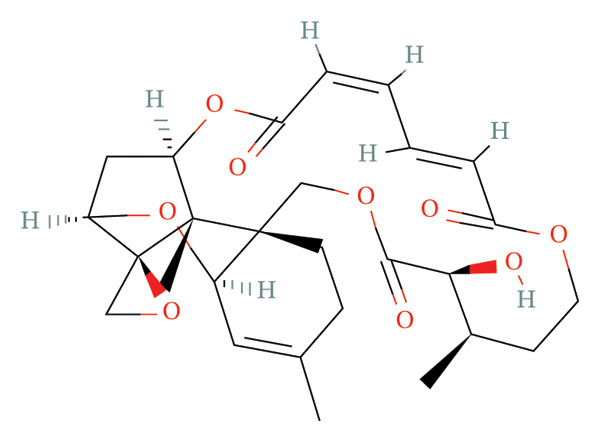
(d)

**Figure:**
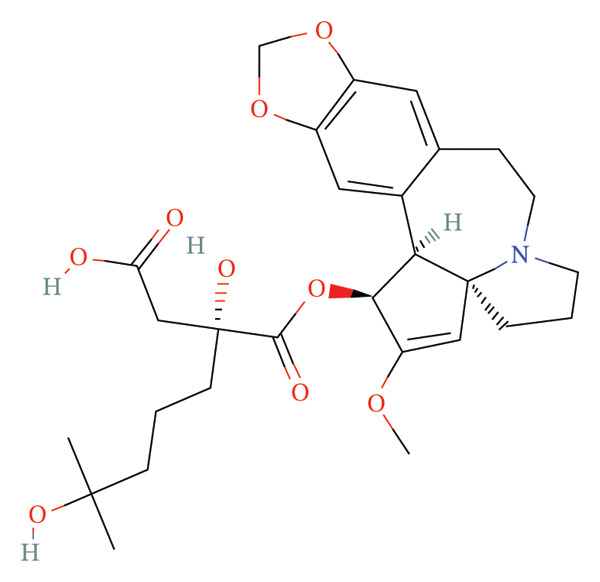
(e)

### 3.8. Drug Prediction for Asthma Targeting

We categorized the 50 most significantly altered genes from both up‐ and downregulated differentially expressed sets into two clusters and performed pharmacological prediction analyses utilizing the Connectivity Map database. Our findings indicated that transcriptional alterations mediated by compounds such as KI‐8751, verrucarin‐a, tyrphostin‐AG‐126, and homoharringtonine exhibited strong inverse relationships with disease‐associated expression patterns, implying potential therapeutic efficacy for disease amelioration (Figures 9(b), 9(c), 9(d), and 9(e)).

## 4. Discussion

This investigation combined transcriptomic datasets, MR approaches, and scRNA‐seq to holistically characterize and verify central genes contributing to asthma development. Our results identified seven genes with plausible causal roles in asthma etiology, including five—DUSP5, FCER1A, LGALS2, SHISA2, and USP36—that showed persistent suppression in asthma cohorts and significant enrichment in immune‐modulating pathways. These observations yield fresh understanding of asthma’s molecular foundations and point to viable targets for therapeutic intervention.

The MR analysis provided robust genetic evidence corroborating the involvement of these genes in asthma. In particular, FCER1A and LGALS2—genes with established roles in immune regulation—were found to be associated with decreased asthma risk [12–14]. Their reduced expression in asthmatic tissues implies a loss of protective capacity during pathogenesis, aligning with prior reports that have linked FCER1A to allergic mechanisms and LGALS2 to inflammatory modulation [15]. Likewise, DUSP5, a modulator of MAPK signaling, may contribute to asthma pathogenesis via its effects on immune cell communication and inflammatory processes [16, 17].

Functional enrichment studies reinforced the participation of these genes in essential immunological mechanisms. Notable enrichments were observed for pathways including IL‐17 signaling, Th1/Th2 differentiation, and NF‐κB activation, highlighting the critical role of immune dysregulation in asthma pathogenesis. These mechanisms represent well‐documented drivers of asthma development, particularly in promoting airway inflammation and structural remodeling [18–22].

Single‐cell profiling enabled precise mapping of cell type–specific expression for these pivotal genes. For instance, FCER1A and LGALS2 showed elevated expression in dendritic cells, which are central to antigen presentation and T‐cell activation, implying their potential involvement in modulating asthma through dendritic cell regulation. Likewise, the pronounced expression of DUSP5 in goblet cells suggests a functional association with mucus secretion and airway hyperreactivity.

The immune infiltration analysis revealed a distinct landscape in asthma samples, characterized by an increase in resting mast cells and decreases in memory B cells, eosinophils, and naïve CD4+ T cells. The correlations between key genes and immune cell subsets suggest that these genes may contribute to asthma by modulating the recruitment and activation of specific immune cells [23, 24]. For instance, the positive correlation between USP36 and memory B cells aligns with its potential role in adaptive immunity.

From a translational medicine standpoint, our computational drug screening revealed several candidate compounds—including homoharringtonine and tyrphostin AG‐126—with potential to counteract asthma‐related transcriptional signatures. These results highlight promising opportunities for drug repositioning and the creation of precision therapeutics for asthma.

Although this research demonstrates considerable merits, several limitations should be acknowledged. First, our MR analysis relied on blood‐derived eQTL data from the eQTLGen consortium. Given the known tissue specificity of eQTLs, the regulatory strength and direction of the identified SNPs on gene expression in peripheral blood may differ from those in asthma‐relevant tissues such as the airway epithelium or lung parenchyma. Although we validated the differential expression of our core genes in independent asthma lung tissue datasets, which mitigates tissue‐context bias to some extent, we cannot confirm that the SNP instruments exert identical regulatory effects in the disease‐relevant microenvironment. This may affect the precision of our causal inference. Second, despite rigorous sensitivity analyses (MR‐Egger and MR‐PRESSO) to detect and correct for pleiotropy, residual horizontal pleiotropy—where SNPs influence asthma risk through pathways independent of the candidate gene—cannot be entirely ruled out. Finally, the biological functions and therapeutic potential of the highlighted genes require further validation through in vitro and in vivo experimental models.

## 5. Conclusion

In conclusion, based on integrative multiomics analysis combining transcriptomic profiling, MR, and scRNA‐seq, this study identifies and validates five key genes—DUSP5, FCER1A, LGALS2, SHISA2, and USP36—as causally associated with asthma risk, revealing their roles in immune dysregulation through pathways including IL‐17 signaling and T‐cell differentiation. These genes demonstrate cell type–specific expression in airway environments and correlate with altered immune infiltration features, providing mechanistic insights into asthma pathogenesis. Furthermore, connectivity mapping suggests candidate therapeutic agents capable of reversing disease‐associated gene expression, highlighting potential targets for future asthma therapeutics.

## Author Contributions

Chao Yuan and Shuman Li designed this work. Fang Liu, Hongtao Cui, and Yan Mei performed the analyses, visualization, and manuscript editing. Keyu Li and Yan Xu performed the data analyses.

## Funding

This study was supported by the Science and Technology Research Program of Chongqing Municipal Education Commission (Grant Nos. KJQN202315133, KJQN202415133, and KJQN202415144); special funding for postdoctoral research project of Chongqing Municipal Human Resources and Social Security Bureau (2022CQBSHTB1029 and Document No. 298 [2019]); and the Natural Science Foundation of Chongqing (cstc2021jcyj‐msxmX0505 and cstc2022jxjl120015).

## Disclosure

All the authors have read and approved the final version of the manuscript.

## Ethics Statement

The authors have nothing to report.

## Consent

The authors have nothing to report.

## Conflicts of Interest

The authors declare no conflicts of interest.

## Supporting Information

Additional supporting information can be found online in the Supporting Information section.

## Supporting information


**Supporting Information** Supporting Figure 1: Single‐cell preprocessing.

## Data Availability

Any data generated in the analysis process can be requested from the corresponding author.
