# Supplementary material for: Multiomics Approach Identifies Novel Genetic Determinants and Therapeutic Targets for Asthma
Source: Can Respir J. 2026 May 14;2026:6447989. doi: 10.1155/carj/6447989 (PMC13175393; doi:10.1155/carj/6447989)
Supplement: Supplementary file 1 — Supporting Information Supporting Figure 1: Single‐cell preprocessing. [file CARJ-2026-6447989-s001.docx]

**Multi-Omics Approach Identifies Novel Genetic Determinants and Therapeutic Targets for Asthma**


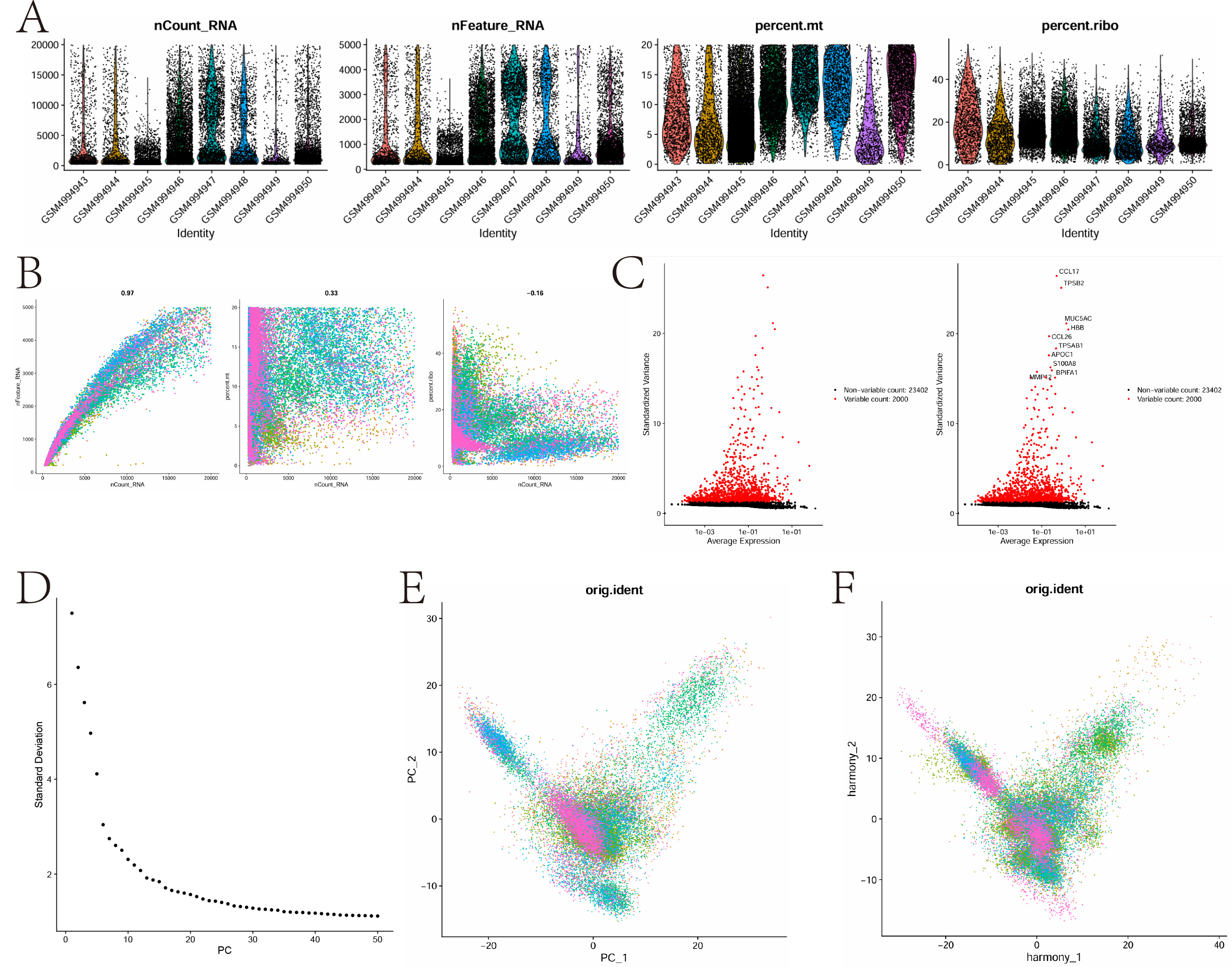


**Fig. S1: Single-cell Preprocessing**

(A) Single-cell quality control, showing the number of cells, genes, and sequencing depth for each sample. (B) Left: relationship between cell sequencing depth and mitochondrial content; Middle: relationship between mitochondrial content and nCount_RNA; Right: relationship between sequencing depth and number of genes. Scatter plots illustrate the correlation between mitochondrial content (y-axis) and nCount_RNA (x-axis). Each dot represents a cell, demonstrating the distribution of RNA counts relative to mitochondrial gene expression levels. (C) Identification of genes with significant variation across cells, accompanied by a feature variance plot. (D) Variance ranking plot for each principal component (PC). (E-F) Visualization of PCA results and distribution of PCs, where dots represent cells and colors represent samples.
